# Predictive accuracy of fecal calprotectin in assessing clinical activity and disease severity in patients with Ulcerative Colitis and Crohn’s disease

**DOI:** 10.1186/s12876-025-04035-2

**Published:** 2025-06-04

**Authors:** Ankit V. Jain, Sandeep Gopal, Anurag J. Shetty, Suresh Shenoy, B. V. Tantry, B. Unnikrishnan, Ramesh Holla, Rishit Anand

**Affiliations:** 1 Department of Medical Gastroenterology, Kasturba Medical College Mangalore, Manipal Academy of Higher Education, Manipal, Karnataka 576 104 India; 2 Department of Community Medicine, Kasturba Medical College Mangalore, Manipal Academy of Higher Education, Manipal, India; 3 Kasturba Medical College Mangalore, Manipal Academy of Higher Education, Light House Hill Road, Mangalore, Manipal, Karnataka 575001 India

**Keywords:** Fecal calprotectin, IBD, Crohn disease, Ulcerative colitis

## Abstract

**Background:**

Inflammatory bowel disease (IBD) is an idiopathic disorder characterized by repeated relapses and remissions. Endoscopy, the gold standard for diagnosis and monitoring of these patients is time consuming, expensive, and invasive. Hence, faecal calprotectin (FCP) has been suggested as marker to determine the degree of intestinal inflammation and predict relapse in IBD.

**Objective:**

To use FCP as a predictor of clinical activity and endoscopic severity in IBD patients in a tertiary care hospital in Southern India.

**Methods:**

Study subjects underwent clinical examination, endoscopy, blood tests and stool FCP. For Endoscopic activity simple endoscopic score for Crohn’s disease (SES-CD) and Ulcerative Colitis endoscopic index of severity (UCEIS) scores were used, and clinical activity was assessed by Crohn’s disease activity index (CDAI) and simple clinical colitis index (SCCAI) for CD and UC respectively. At six months, blood, and stool FCP test were repeated which were compared with endoscopic and clinical activity indices.

**Results:**

The number of males was higher in both CD (13/8) and UC (19/14). At first visit and follow up, CDAI and FCP were positively correlated (r-0.689, p- 0.016) (r- 0.425, p-value < 0.05). In CD, the sensitivity and specificity of FCP in detecting active disease and remission were 93.8% and 80% respectively (AUC-0.869). At follow up, the sensitivity and specificity were 80% and 93.3% respectively (AUC-0.867). In patients with UC, SCCAI score and FCP levels positively correlated (r-0.231/0.387, p-value 0.001/0.001) at both the first and follow up visits. The sensitivity of FCP in detecting UC in active and remission states was 92.6% whereas the specificity was 83.3%. AUC was 0.88. At the time of follow up, the sensitivity of FCP in detecting UC in active and remission states was 89.9% whereas the specificity was 87.0% and AUC was 0.879.

**Conclusion:**

This study confirmed that FCP level shows strong association with clinical and endoscopic activity indices in patients of IBD. Therefore, FCP levels could be used as a surrogate marker for monitoring mucosal status as well as predicting endoscopic remission in IBD patients.

## Introduction

Inflammatory bowel disease is a disorder characterized by repeated relapses and remissions [1]. According to an IBD burden estimate, there are 0.27 million cases in India in 2019, almost double the number in 1990–0.13 million [2]. In resource constrained settings, there are still challenges in diagnosis, evaluating disease activity and severity, prognostication, and treatment.

The most common method for monitoring patients is with IBD is endoscopy. multiple grading systems are available to monitor endoscopic activity in IBD. The most often utilized are the basic endoscopic grading in Crohn’s disease and the Ulcerative Colitis endoscopic index of severity [3–5]. Endoscopy is a time consuming, expensive, and invasive procedure. Moreover, intestinal lavage prior to endoscopic procedure is uncomfortable for patients. Given these facts, non-invasive markers for measuring disease activity in IBD patients would be an invaluable.

The measurement of disease activity is critical for monitoring response and guiding treatment. While invasive and expensive, lower gastrointestinal endoscopy and histology of tissue biopsy samples is the gold standard both for diagnosing IBD and for follow up of the disease. Despite this, researchers have been attempting to identify a non-invasive test (NIT) for IBD for several reasons. Firstly, as symptoms do not always accurately reflect disease activity an objective measure of disease activity is required. Secondly, an accurate NIT would spare the patient the stress and discomfort of an invasive procedure [6]. The ideal marker would be easy to administer, quick to complete, affordable, and would have results which are consistent across patients and laboratories. It would also bespecific to the disease in question and would perhaps be able to identify people at risk for the illness as well. It would be able to identify signs of disease activity and determine the effectiveness of treatment. It would add diagnostic information too [6]. So far, the commonly used NIT tests - CRP and ESR - are sensitive or specific enough [7]. Calprotectin, a faecal marker that is secreted by myelomonocytic cells, has antibacterial and anti-proliferative properties, and regulates the inflammatory process [8, 9]. Calprotectin is secreted into the GIT during mucosal inflammation and is a useful marker for detecting intestinal inflammation. It has been proposed that fecal calprotectin could as a non-invasive test monitoring patients with IBD [10–15].

Published literature from India about the utility of fecal calprotectin as an NIT to monitor IBD is scanty. Hence in this study we aimed to determine the utility of faecal calprotectin to measure the clinical and endoscopic disease severity in IBD.

## Objectives

This study aimed to determine whether there a correlation exists between fecal calprotectin levels and clinical activity / endoscopic disease severity in patients with Crohn’s disease or ulcerative colitis.

## Materials and methods

This was a prospective observational study conducted at the department of Gastroenterology, Kasturba Medical College Hospital, Mangaluru, between January 2022 and March 2023. The subjects were adults diagnosed with Crohn’s disease (CD) or ulcerative colitis (UC) based on accepted clinical, radiologic, endoscopic, and histopathological criteria. Patients with indeterminate colitis, use of NSAIDs, confirmed CMV co-infection, Clostridium difficile infection, acute infective diarrhoea, and HIV infection were excluded.

The study received approval from the ethics and scientific committee, and data was collected using a prevalidated proforma. Demographic information, clinical examinations, blood tests, and fecal samples for calprotectin levels were obtained from enrolled patients. Disease severity was assessed through endoscopic procedures and clinical activity indices using specific scoring systems for UC (UCEIS and SCCAI) and CD (SES-CD and CDAI). Standard therapy was offered to all patients, and a six-month follow-up included repeat assessments and endoscopy as needed. The study aimed to analyse fecal calprotectin levels in relation to clinical activity and endoscopic severity in patients with UC and CD.

### Statistical analysis

Statistical analysis was done using Statistical Package for the Social Sciences- SPSS version 29. The collected data was coded and entered in SPSS software for analysis. Quantitative data was described in the form of mean and standard deviation; qualitative data was described in the form of frequency and percentage.

Spearman Correlation was used to analyse the correlation of fecal calprotectin with SES CD and CDAI in patients with Crohn’s disease, UCEIS and SCCAI in patients with Ulcerative Colitis, reliability of Fecal calprotectin in diagnosing Crohn’s disease and Ulcerative colitis was analysed by sensitivity and specificity. P-value of less than 0.05 was considered as statistically significant for all tests.

### Observations and results

Fifty-four patients were included in the study after applying all the selection criteria. Twenty-one patients had CD and 33 patients had UC. Of these 54 patients, one patient of CD was lost for follow up and one patient of UC died due to acute severe disease during the hospital stay. Male preponderance was seen in both CD (13 male and 8 female patients) and UC (19 male and 14 female patients). Most patients were in their 3rd to 4th decade in CD (15 out of 21) and 4th to 6th decade in UC (25 out of 33). For CD, 10 patients were in age group of 31–50 years and only 1 patient was above 50 years of age whereas for UC, 8 patients were in age group of 20–30 years followed by 9 patients in the age group of 31–50 years and 16 patients above the age of 50 years.

Based on the clinical activity index (CDAI), 14 patients were clinically active with mild, moderate, and severe activity present in 1, 12 and 1 respectively. Seven patients were in remission. On follow up, 4 patients had active disease (1 in mild and 3 in moderate) and 16 patients were in remission. In patients of CD based on the endoscopic score (SES-CD), 16 patients were active whereas 5 patients were in remission at first visit. In patients of active disease mild, moderate, and severe activity was present in 2, 8 and 6 patients respectively. At follow up, five patients were active (2 had mild and 3 had moderate disease activity) while majority of patients were in remission (15 out of 20 patients were in remission).

### SES-CD and its correlation with ESR, CRP and FCP

We compared SES-CD and FCP for CD patients at the time of first visit and found that in all patients except one, FCP was low when the disease was inactive. Patient with active disease tended to have high FCP levels. When SES-CD was above 2 all the patients had high FCP. Spearman correlation showed statistically significant correlation with p-value < 0.001 for first visit for FCP. In correlation between SES-CD and FCP for follow up visits, the study reported most of the patients in remission, based on the SES-CD had normal FCP value. Few patients with normal SES-CD had raised FCP. One patient with raised SES-CD had borderline FCP. The Spearman correlation coefficient showed positive correlation with statistically significant p-value of < 0.001 for ESR as shown in Table 1. Table 1 shows the Spearman correlation between SES-CD and ESR, CRP and FCP with p-value.

**Table 1:** Correlation between SES-CD score and ESR, CRP and FCP of study subjects with Crohn’s disease at the time of their first visit and follow-up visit (*n* = 21)

First Visit	Spearman Correlation coefficient	*P*- value
ESR	0.253	0.269
CRP	0.480	0.028
FCP	0.827	< 0.001
**Follow-up Visit**		
ESR	0.799	< 0.001
CRP	0.151	0.525
FCP	0.483	0.031

### CDAI and its correlation with FCP

The clinical activity of CD patients was assessed using CDAI scoring system at the time of their first visit and the values of CDAI score and FCP were compared. The mean CDAI score was 239.42 and the mean FCP value was 561.79. The correlation was analysed using Spearman correlation test and it showed that CDAI score and FCP values were positively correlated with the correlation coefficient of 0.859. The correlation was statistically significant for FCP with p- value of < 0.001 as shown in Table 2. At follow up the correlation between CDAI score and FCP was studied; the mean CDAI score was 153.95 and the mean FCP was 163.46. The correlation was analysed using Spearman correlation test which showed that CDAI score and FCP levels positively correlated with correlation coefficient of 0.304 and p-value of 0.193 as shown in Table 2.

**Table 2:** Correlation between score CDAI score and FCP of study subjects with Crohn’s disease at the time of their first visit and follow-up visit

At first visit					
**Investigation**	**N**	**Mean**	**Standard Deviation**	**Spearman Correlation coefficient (r)**	**p-value**
CDAI Score	21	239.42	77.916		
FCP (µg/g)	21	561.79	460.747	0.859	< 0.001
**At follow-up visit**					
CDAI Score	20	153.95	74.101		
FCP	20	163.46	263.775	0.304	0.193

**Graph. 1:**
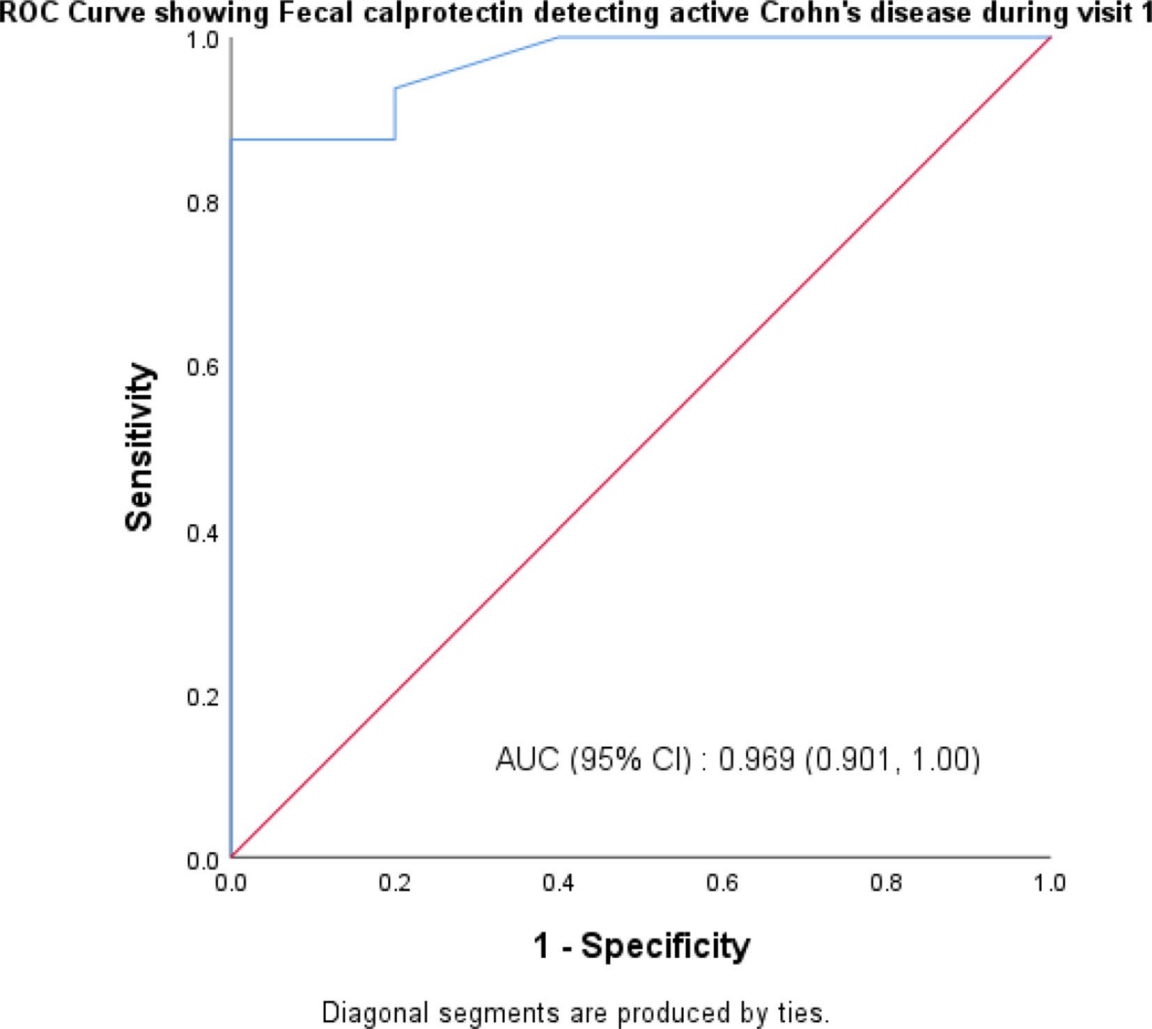
ROC Curve showing Sensitivity and Specificity of fecal calprotectin in detecting Crohn’s disease in active and remission form on first visit

**Graph. 2:**
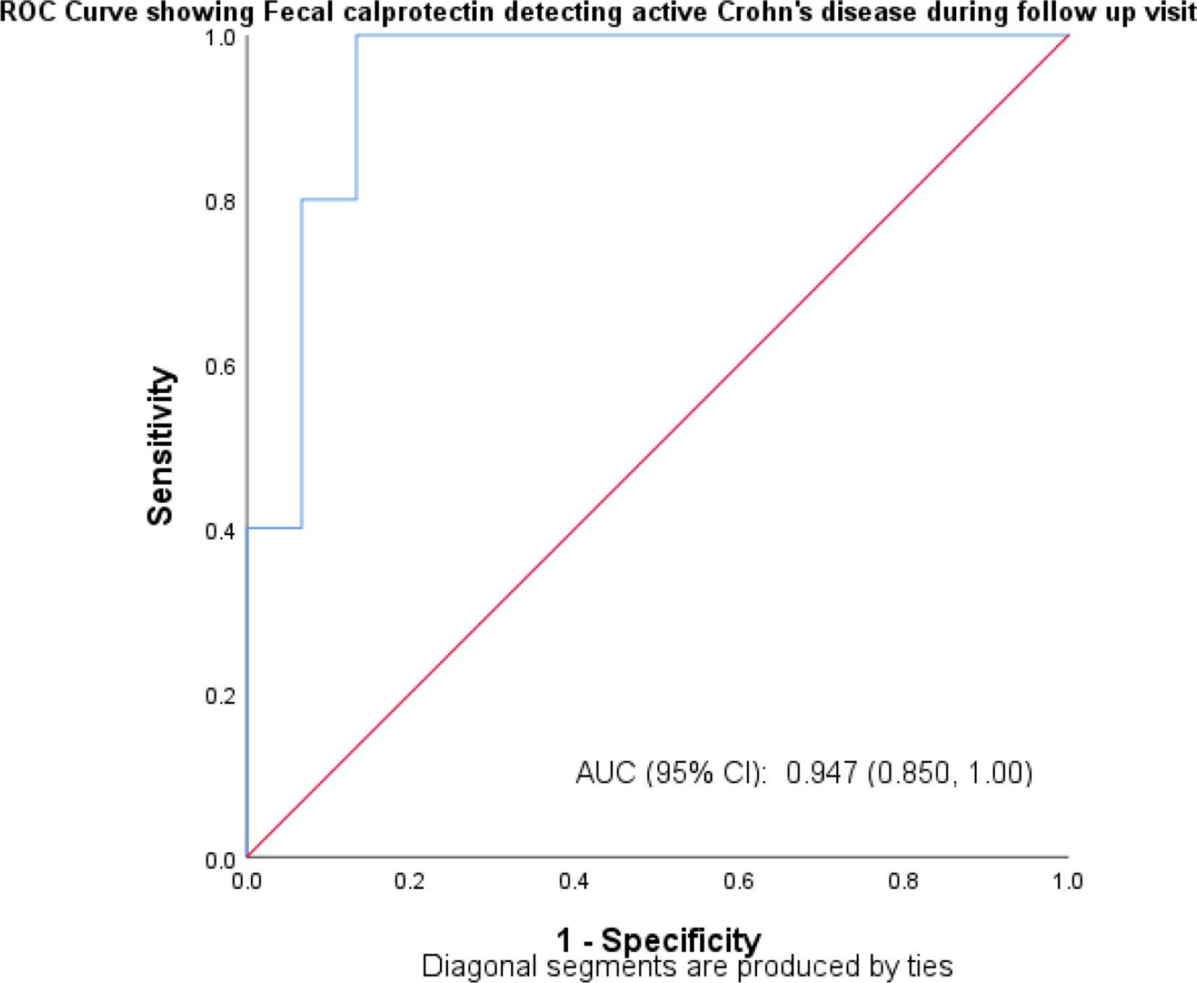
ROC Curve showing Sensitivity and Specificity of fecal calprotectin in detecting Crohn’s disease in active and remission form on follow-up visit

The sensitivity and specificity of FCP in identifying CD in active and remission form in patients at their initial visit were 93.8% and 80%, respectively, with an area under the curve of 0.867. At follow-up, FCP had an 80% sensitivity and a 93.3% specificity in identifying CD in active and remission forms with an area under the curve of 0.869, respectively as shown in Table 3. Graph 1 shows the area under the curve, which was 0.867 for first visit and Graph 2 shows the under the curve, which was 0.869 for follow-up visit.

**Table 3:** Sensitivity and specificity of fecal calprotectin in detecting Crohn’s disease in active and remission form at first and follow up visit

Crohn’s disease first visit		Fecal Calprotectin (µg/g)	
		Active (> 50)	Remission (< 50)
SES-CD	Active (> 2)	15	1
	Remission (< 2)	1	4
Sensitivity	93.8%		
Specificity	80%		
Area under curve	0.867		
**Crohn’s disease follow up visit**			
SES-CD	Active (> 2)	4	1
	Remission (< 2)	1	14
Sensitivity	80%		
Specificity	93.3%		
Area under curve	0.869		

In patients of UC, blood in stools was present in 97% whereas loose stools were present in 84.8% of patients. On follow up, blood and loose stools were present in 18.8% and 28.1% respectively. Based on the SCCAI clinical activity index, 25 patients were active whereas 8 patients were in remission. On follow up, 9 patients had active disease and 23 patients were in remission. Based on endoscopic activity index (UCEIS) active disease was seen in 26 out of 33 patients of which 4, 17 and 5 patients had mild, moderate, and severe form of disease. On follow up, 11 patients had active disease with mild, moderate, and severe disease activity seen in 7, 3 and 1 patient and majority of patients were in remission (21 out of 32 patients).

### UCEIS and its correlation with ESR, CRP and FCP

On correlation between UCEIS and FCP in patients of UC at the time of first visit, the study observed that majority of patients with high UCEIS score had high FCP showing very strong correlation. Only two patients with high UCEIS had normal FCP. On applying Spearman correlation, a positive correlation was seen with statistically significant p value as shown in Table 4.

On correlation between UCEIS and FCP in patients of UC at the time of follow up, the study reported that majority of patients were in remission and had normal FCP value. Three patients with high UCEIS had normal FCP. On applying Spearman correlation, it was found that FCP and UCEIS were positively correlated with statistically significant p-value. Table 4 shows Spearman correlation between UCEIS and its correlation with ESR, CRP and FCP.

**Table 4:** Correlation between colonoscopy UCEIS score and ESR, CRP and FCP of study subjects with ulcerative colitis at the time of their first visit and follow-up visit

First Visit	Spearman Correlation coefficient	*P*- value
ESR	0.064	0.704
CRP	0.311	0.09
FCP	0.793	< 0.001
**Follow-up Visit**	**Spearman Correlation coefficient**	**P- value**
ESR	0.305	0.090
CRP	0.505	0.003
FCP	0.369	0.037

### SCCAI and its correlation with FCP

Table 5 shows the correlation between SCCAI score and FCP levels in UC at the time of their first visit. The mean SCCAI score was 6.63 and the mean FCP value was 765.79. SCCAI score and CP levels positively correlated as analysed by Spearman correlation test, the correlation coefficientwas 0.447 and this correlation was statistically significant (p-value 0.009). At follow up, the mean SCCAI score was 2.43 and the mean FCP value was 162.05. SCCAI score and FCP levels in UC patients was found to correlate positively as analyzed by Spearman Correlation.. The correlation coefficient was 0.426 and this correlation was statistically significant (p-value 0.015) as shown in Table 5.

**Table 5:** Correlation between SCCAI score and fecal calprotectin of study subjects with ulcerative colitis at first and follow-up visits

**At first visit**					
**Investigation**	**N**	**Mean**	**Standard Deviation**	**Spearman Correlation coefficient (r)**	**p-value**
SCCAI score	33	6.636	2.876	0.447	0.009
Fecal calprotectin Value	33	765.79	171.522		
**At follow-up**					
SCCAI score	32	2.43	2.711	0.426	0.015
Fecal calprotectin Value	32	162.05	290.101		

**Graph. 3:**
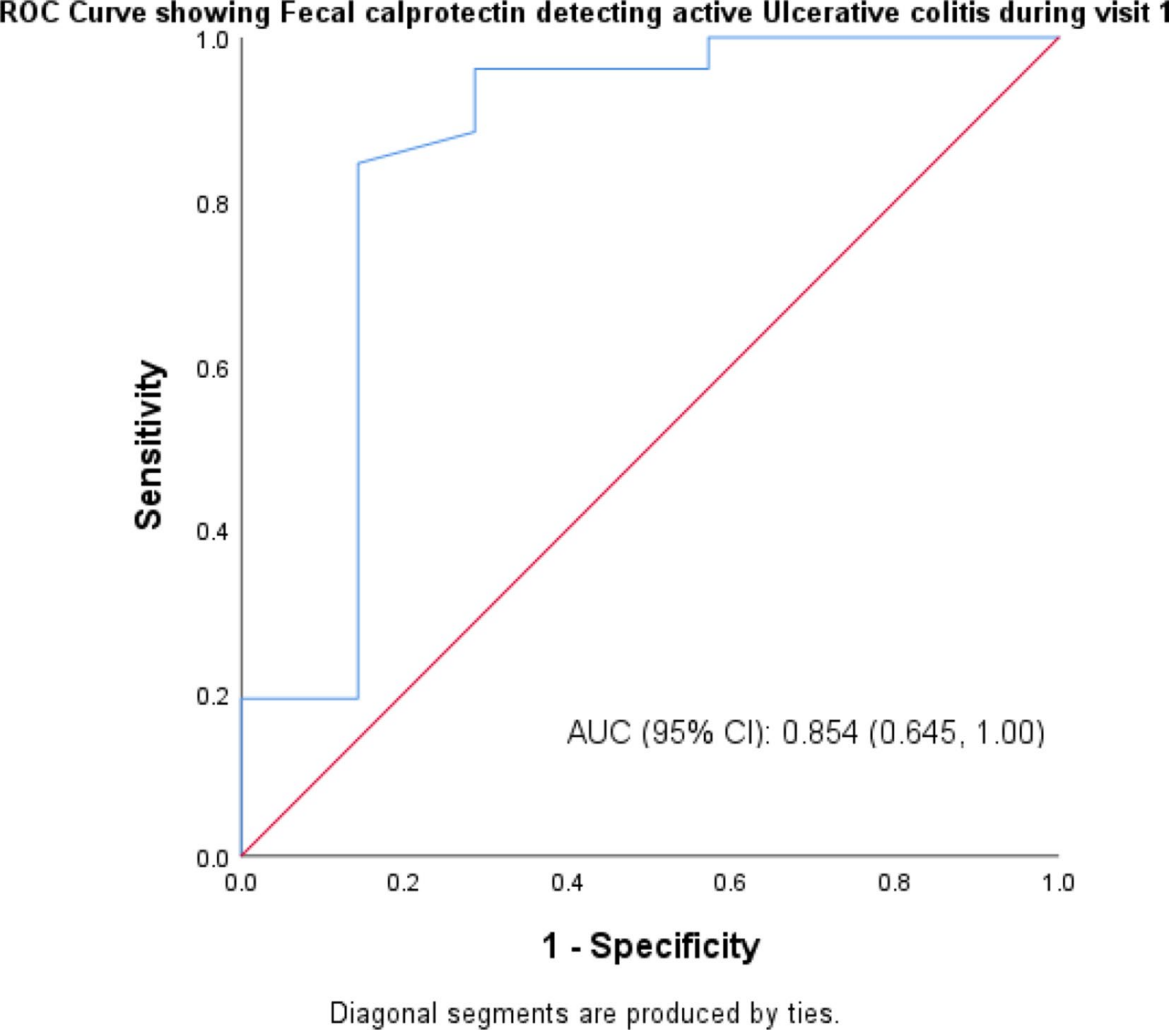
ROC curve depicting sensitivity and specificity of fecal calprotectin in detecting UC at the time of first visit

**Graph. 4:**
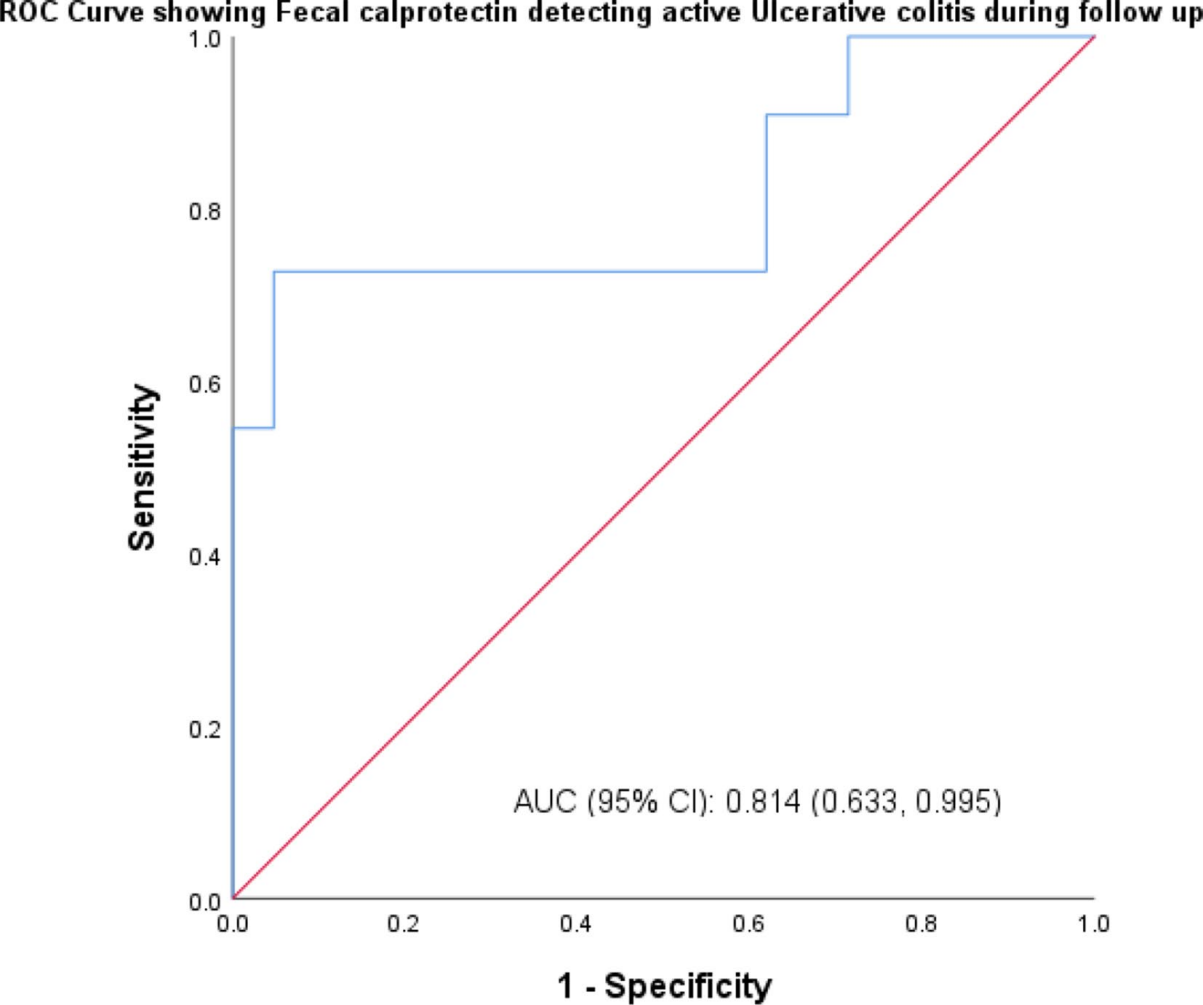
ROC curve depicting sensitivity and specificity of fecal calprotectin in detecting UC at the time of follow-up visit

The sensitivity of FCP in identifying UC in both active and remission form was 92.6% at the time of the patient’s initial visit, while the specificity was 83.3% and the area under the curve was 0.88. At the time of follow-up, the specificity was 87.0%, the area under the curve was 0.879, and the sensitivity of FCP in diagnosing UC in both active and remission form was 89.9% as shown in Table 6. Graph 3 represents area under curve for UC at initial visit being 0.88 and Graph 4 represents area under curve for UC at follow-up visit being 0.879.

**Table 6:** Sensitivity and specificity of fecal calprotectin in detecting ulcerative colitis at first and follow up visit

Ulcerative Colitis first visit		Fecal Calprotectin (µg/g)	
		Active (> 50)	Remission (< 50)
UCEIS	Active (> 1)	25	1
	Remission (< 1)	2	5
Sensitivity	92.6%		
Specificity	83.3%		
Area under curve	0.88		
Ulcerative Colitis follow up visit		**Fecal Calprotectin (µg/g)**	
		Active (> 50)	Remission (< 50)
UCEIS	Active (> 1)	8	3
	Remission (< 1)	1	20
Sensitivity	89.9%		
Specificity	87.0%		
**Area under curve**	0.879		

## Discussion

Inflammatory bowel diseases are chronic diseases of the digestive tract, with periods of remission and relapses. Despite recent improvements in IBD diagnosis and treatment, a simple, non-invasive, reliable, and affordable way to gauge disease activity and track therapeutic response is needed. Lower gastrointestinal endoscopy, the gold standard method, is invasive and has a small but definite risk of complications. Currently, most of the research indicates that endoscopic and/or histological examination is the most effective method for assessing inflammation [16, 17]. Although subjective and frequently impacted by non-inflammatory illness variables such as fibrosis, symptoms can serve as markers of inflammation and disease activity. Many studies have been done in the past on inflammatory markers and its role to determine the disease activity in IBD patients [18, 19]. However, the literature is scarce on the follow up studies done on the patients of IBD. To demonstrate the utility of FCP in assessing disease activity, we measured the FCP levels of UC and CD patients at multiple times (six months) with varied levels of disease activity in the same patient to demonstrate the strong relationship between FCP and disease activity.

Most of the patients included in the study were initially in the active phase of their disease. At follow up, which was done at six months for both CD and UC most of the patients were in the remission. In this study the clinical activity of CD patients was determined using CDAI score. In patients of UC the clinical activity was determined using SCCAI score. Then it was correlated with FCP. According to CDAI clinical criteria, patients with active CD had significantly higher FCP levels than patients in remission. FCP levels were correlated with the CDAI score and the correlation was statistically significant both at first visit and on follow up. Similar observation was made by Lee YW et al. [20] in their study where FCP positively correlated with CDAI. A study conducted by MP Narayanan et al. [21] from south India made similar observations where they concluded that in patients of CD FCP correlated with CDAI. In patients with UC in our study, clinical activity was determined using SCCAI score. It was observed that FCP showed positive correlation with SCCAI score and it was statistically significant. A study from India by Nagesh Kamat et al. [22] made similar observations in their study where they found positive correlation between FCP and SCCAI. Mark Samaan et al. [23] also made similar observations.

In this study it was observed that mean FCP was higher in patients with UC as compared to those with CD. Also, the FCP mean was higher in patients with active disease than in patients who were in remission. There was positive correlation of inflammatory markers with disease severity. Hrishikesh S et al. in their study from India have made similar observations where mean of FCP was more in UC than CD patients. It was more in active disease than in those who were in remission.

Fecal calprotectin had a sensitivity of 93.8% and a specificity of 80% in identifying CD patients in active and remission forms at their initial visit. At follow-up, FCP had an 80% sensitivity and 93.3% specificity in identifying CD in both active and remission forms. When UC patients saw their first doctor, the sensitivity of FCP in identifying both active and remission-state UC was 92.6%, while the specificity was 83.3%. At the time of follow-up, FCP had an 89.9% sensitivity and an 87.0% specificity in identifying UC in both active and remission forms. A recent meta-analysis conducted by T.Rokkas et al. showed that FCP was superior to CRP and ESR in the diagnosis of IBD [16].The findings of the current study also showed direct correlation between FCP level and endoscopic activity indices which is similar to studies done by Schoepfer A et al. and Vieira A et al. studies [24, 25]. Serum and fecal biomarkers were recommended by the STRIDE II trail as feasible intermediate- to medium-term treatment goals, where treatment may be reviewed only based on these tests, to support care in the clinic environment [26]. The FCP test is an easy, repeatable, and non-invasive procedure. The findings of this study showed a substantial correlation between the endoscopic index—which was assessed by the SES-CD for CD and the UCEIS for UC—and the concentration of FCP in IBD patients. Additionally, it showed a substantial correlation between the clinical activity indices, as measured by the CDAI for CD patients and the SCCAI for UC patients, and the concentration of FCP in IBD patients.

This study was limited by the small sample size. The fact that it was a single centre project limits the generalizability. The data was collected during the COVID-19 pandemic where only patients with grave symptoms or severe relapse visited hospital [17] which may have led to a selection bias. Study subjects were offered standard of a care therapy as prescribed by the treating physician. Inter subject variation in treatment may have influenced results.

Future research with larger sample sizes may be required to address some of the shortcomings and further assess the utility of this biomarker in clinical practice.

Despite these limitations, our research confirms that FCP levels are associated with clinical activity and endoscopic severity. By identifying endoscopic recurrence through serial FCP assessments, the imperfect sensitivity of single measures was addressed.

## Conclusions

The findings of this study offered additional proof that there is a strong correlation between FCP and mucosal disease activity. Further investigation may yield information on the value of FCP as a prognostic tool to track mucosal and endoscopic health in individuals with IBD.

## Data Availability

The dataset is only available on a reasonable request from the corresponding author.
